# Seismic hazard assessment for Guinea, West Africa

**DOI:** 10.1038/s41598-022-06222-7

**Published:** 2022-02-16

**Authors:** Stephen A. Irinyemi, Domenico Lombardi, Syed M. Ahmad

**Affiliations:** 1 Department of Mechanical, Aerospace and Civil Engineering, University of Manchester, Manchester, UK; 2 Centre for Crisis Studies and Mitigation, University of Manchester, Manchester, UK

**Keywords:** Natural hazards, Engineering

## Abstract

Guinea is located on a stable continental region in West Africa, this is a region characterised by infrequent seismic events. In this study, the seismic hazard level of Guinea and 10 main cities was determined by a probabilistic approach. The calculation was carried out for 10%, 2% and 0.5% probability of exceedance in 50 years (corresponding to 475, 2475 and 9975 years return periods, respectively). We developed a homogenized 100-year catalogue compiled from different seismic sources. Two ground motion prediction equations, originally developed for Eastern and Central North America, a stable continental region, were used for the hazard calculation. A uniform *b*-value of 0.70 ± 0.12, and individual activity rate (λ) were calculated for the three seismic zones considered in this study. The estimated seismic hazard was high in the Palaeozoic area of Guinea. The PGA values estimated for the study region, considering hard rock conditions, were 0.08 g, 0.27 g, and 0.57 g for 475, 2475 and 9975 years return periods, respectively. The results of this study an inform future programmes in disaster risk management and planning for new regional infrastructure.

## Introduction

Guinea is located in West Africa far away from any known active plate boundary. Guinea is not known to be seismically active, and available records suggest that the occurrence of moderate to large earthquakes is infrequent. The first reported earthquake in Guinea occurred in 1795, with an estimated surface magnitude of 5.2, causing considerable damage in the city of Labé^1^. In 1818 an earthquake of surface magnitude, *M*_S_ 5.9 occurred in the Futa Djallon massif in northern Guinea. Another earthquake, with a surface magnitude of 4.0, caused panic in the Kakulima region but no damage was reported. In 1928 an earthquake with a surface magnitude of 4.8 struck the western part of Guinea, leading to the collapse of dwellings along the Konkouré river. Aftershocks followed this event triggering a landslide^1^. More earthquakes were reported between 1935 and 1939. On 22nd December 1983, north-western Guinea experienced a strong earthquake of moment magnitude, *M*_w_, 6.3. The epicentre of the event was located in Gaoual, close to the border of Guinea-Bissau. It resulted in approximately 10 km of surface rupture, which extensively damaged buildings, killing over 300 people and destroying more than 4,000 houses^2,3^. Despite this history of seismicity, no seismic hazard assessment has been carried out for Guinea.

In this study, we develop a seismic source model for the study region using available seismicity and geological information. The ground motion model is constructed using two different strong-motion attenuation equations. Finally, the hazard estimates for the region and ten cities are computed at different return periods: 475, 2475, and 9975 years. The present study provides valuable data for risk assessment and mitigation interventions, land use management, and planning for present and future infrastructures across the study region.

## Geology of Guinea

**Figure 1:**
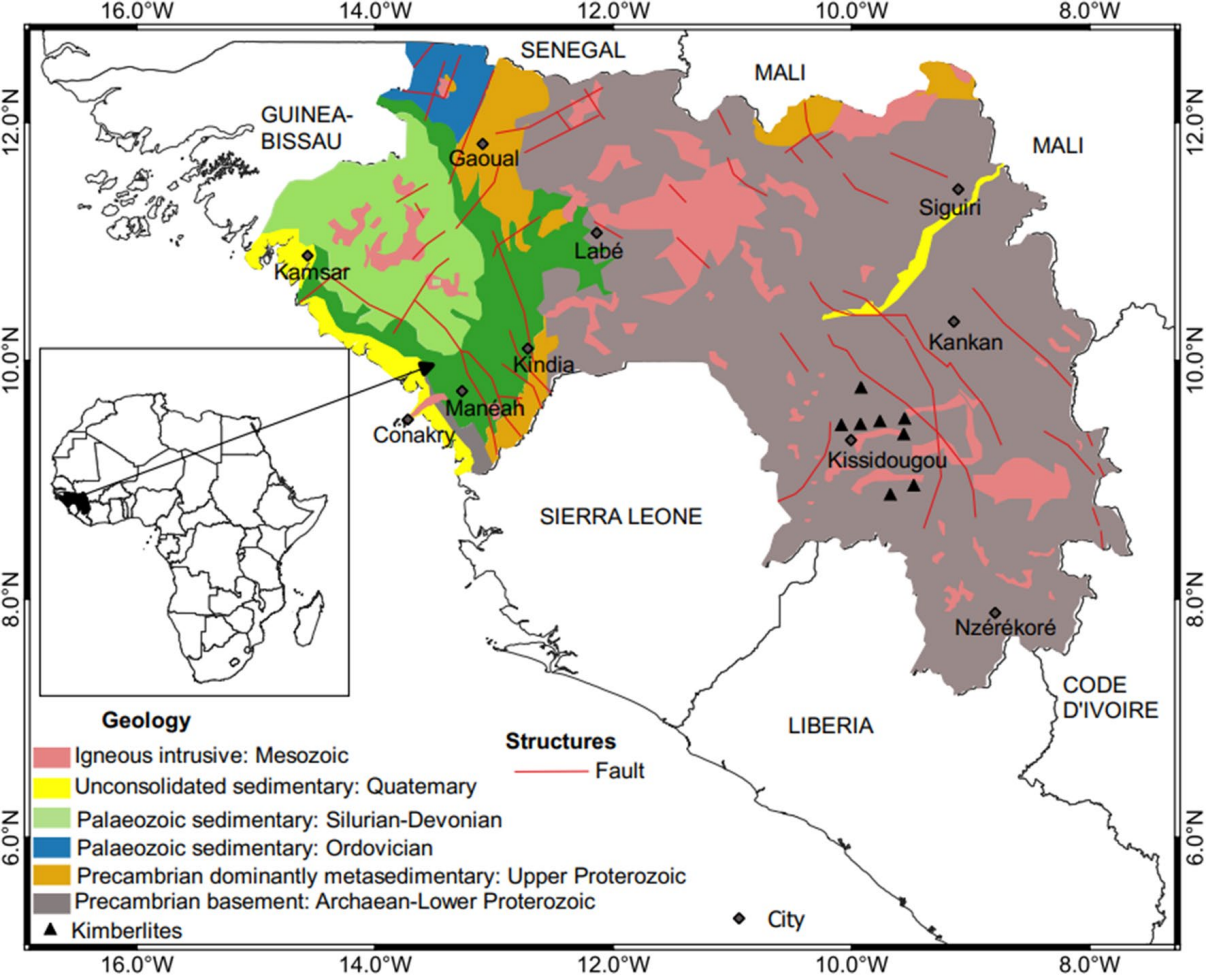
Geology and tectonic map of Guinea.

Guinea has a total surface area of 245,000 km^2^. Figure 1 shows the seismo-tectonic and geology of the study region. This is formed by Precambrian crystalline and Palaeozoic Rocks, which spread along the Guinean-Liberian shield. The Fouta Djallon massif is made of Silurian shade. Ordovician sandstone experienced massive arrival in both dolerites' tertiary and a parent rock gigantic bauxitic with laterite deposits^4^.The northwest of the basin's coastal zone consists of an unconsolidated-small outcrop of upper cretaceous to Tertiary sedimentary rocks. The Mesozoic contains some Kimberlite dykes and pipes located in the southern area that is diamond-bearing^5^. The Western part of the African plate moves at relatively slow rates within 2.0–15 mm/year^6^. The eastern part is primarily underlain by Archaean and Lower Proterozoic rocks, while upper Proterozoic metasedimentary rocks dominate the north. The coastal plains were formed mainly by Quaternary marine and unconsolidated alluvial sediments. Older Palaeozoic overlay the plain, with small Tertiary and Upper Cretaceous sedimentary rocks^2^ explained the faults mechanism that resulted in the 1983 northwest Guinea earthquake recorded in the study. Rocks in Guinea are affected by rokelide orogeny like the one in Sierra Leone deformed during the Pan-African tectonothermal^5^.

## Compiled earthquake catalogue

The historical data sources used for this study were compiled by Ambraseys and Adams^1^ and include events as far back as 1795. The instrumental earthquakes are obtained from international agencies, including: the USGS online catalogue (https://earthquake.usgs.gov/earthquakes/search/) and the International Seismological Centre (ISC) (http://www.isc.ac.uk/iscbulletin/search/catalogue/). We unify the different magnitude scales used in the historical and instrumental database into a single moment magnitude scale (*M*_w_) using the empirical equations given in 1 to 3^7,8^

1 $$M_{w} = 0.85m_{b } + 1.03\; Value\; for\;3.5 \le m_{b } \le 6.2$$

2 $$M_{w} = 0.67M_{s } + 2.07\; Value\; for\;3.0 \le M_{s} { } \le 6.1$$

3 $$M_{w} = 0.97M_{L } + 0.58\; Value\;for\; 3.0 \le M_{L} { } \le 6.0$$

where *M*_L_ = local magnitude (*M*_L_), *M*s = surface magnitude, and *m*_b_ = body magnitude.

**Figure 2:**
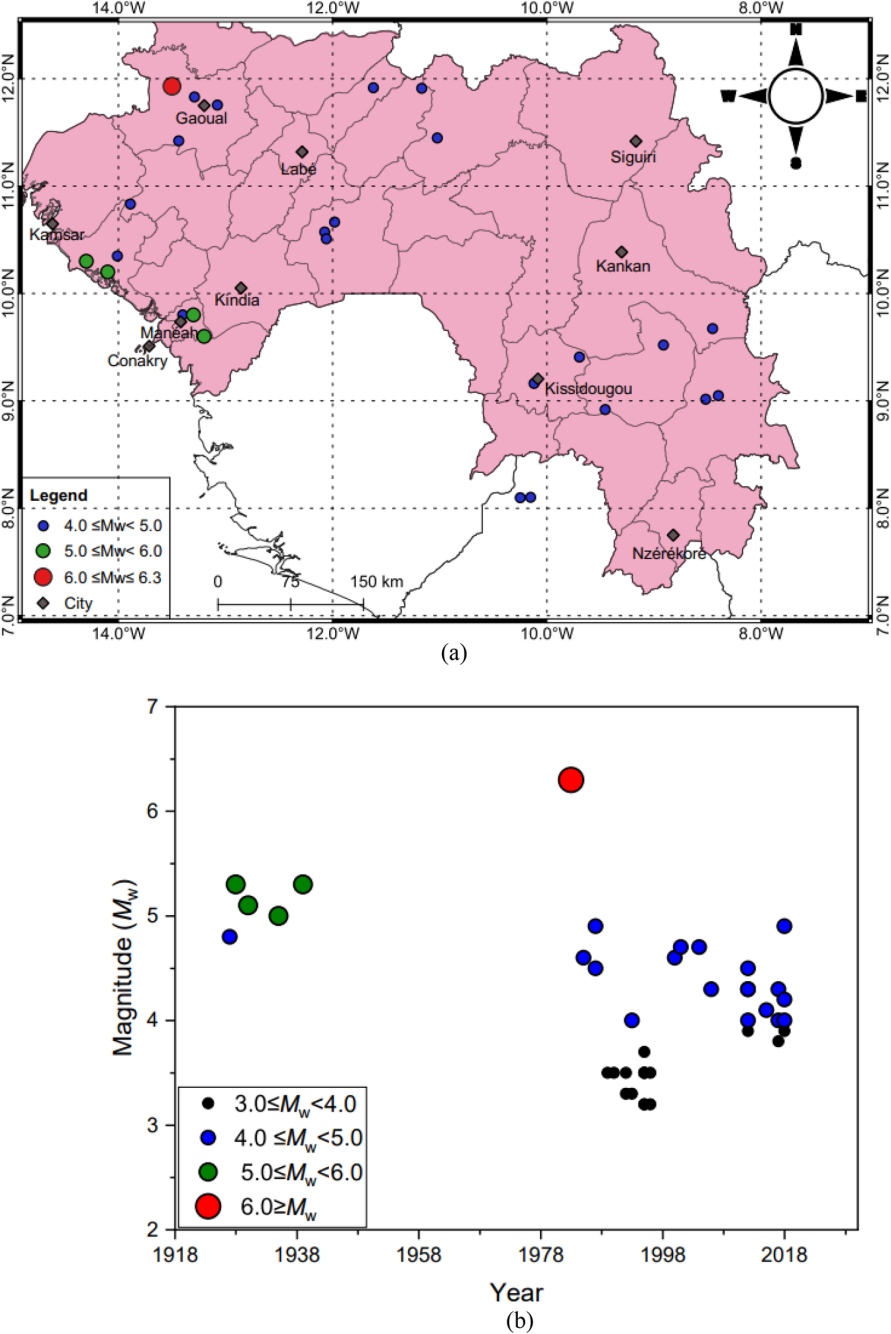
(**a**) Spatial distribution of earthquake epicentres (**b**) Time- magnitude distribution of the *M*_w_ ≥ 3.0 for the period 1918–2018.

As we compile the catalogue from different sources, duplicate events, defined as events with longitude and latitude within 10 km and recorded within a period of 2 minutes^9^ are manually removed. When removing duplicate events, we prioritise events in the ISC earthquake catalogue, followed by the USGS-NEIC earthquake catalogue, and finally in the catalogue by Ambraseys and Adams^1^. To ensure that the earthquakes in the final compiled catalogue are independent events, we remove all foreshocks and aftershocks using the declustering technique by Gardner and Knopoff^10^ which is implemented in the code ZMAP^11^. The compiled catalogue ranges from 4 ≤ *M*_w_ ≤ 6.3. The spatial and temporal distributions of the harmonised and declustered catalogue are shown in Fig. 2. Figure 2a was generated in QGIS Ver. 3.18.3 (https://www.qgis.org/en/site/).

## Seismic hazard analysis

We use the standard probabilistic framework method^12,13^ to estimate the seismic hazard for the study region. We first compute the distribution of recurrence parameters for the events in the harmonised declustered catalogue and use the Stepp method^14^ to assess the completeness of the catalogue. The process assumes that the magnitude sub-class represents a point process in time and follows a Poisson distribution. The unbiased mean rate of occurrence per unit time interval is given by,

4 $$\overline{\lambda } = \frac{1}{N}\mathop \sum \limits_{i = 1}^{N} \lambda_{i}$$

5 $$\sigma_{\lambda } = \sqrt {\overline{\lambda }/T}$$

where λ_i_ = rate of occurrence of events per unit time interval for each subclass magnitude, *N* = number of sub-classes, σ_λ_ = standard deviation and *T* = magnitude class time interval.

**Figure 3:**
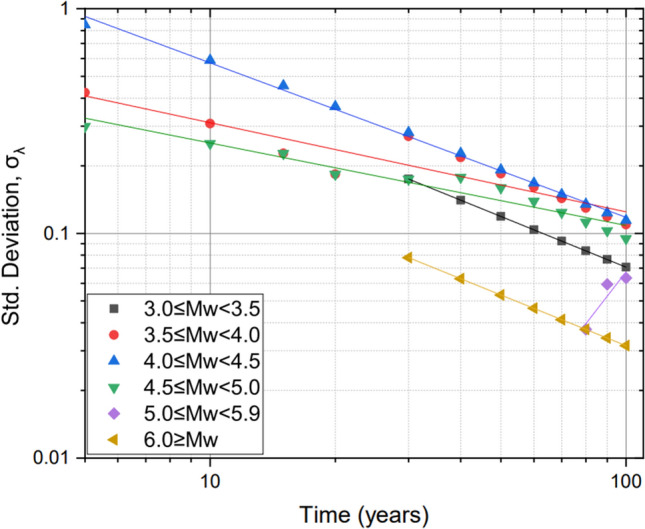
Catalogue completeness periods.

Table 1 and Fig. 3 show the results from the completeness analysis, confirming that the catalogue is complete for *M*w ≥ 4 between 1918 and 2018. Thus, 27 events having event magnitude *M*_w_ ≥ 4.0 were left on the compiled catalogue. The catalogue is complete for *M*_w_ ≥ 3.0 for the period 1978–2018, for *M*_w_ ≥ 4.0 the complete catalogue from 1948–2018 (Table 1). The approach developed by^15^ determined the completeness of the catalogue with respect to time.

**Table 1:** Catalogue completeness for different magnitude sub-classes.

Magnitude sub-class	Period of completeness	Interval (years)
*M*_w_ ≥ 3.0	1978–2018	30
*M*_w_ ≥ 3.5	1948–2018	70
*M*_w_ ≥ 4.0	1948–2018	70
*M*_w_ ≥ 4.5	1948–2018	70
*M*_w_ ≥ 5.0	1918–2018	100
*M*_w_ ≥ 6.0	1918–2018	100

**Figure 4:**
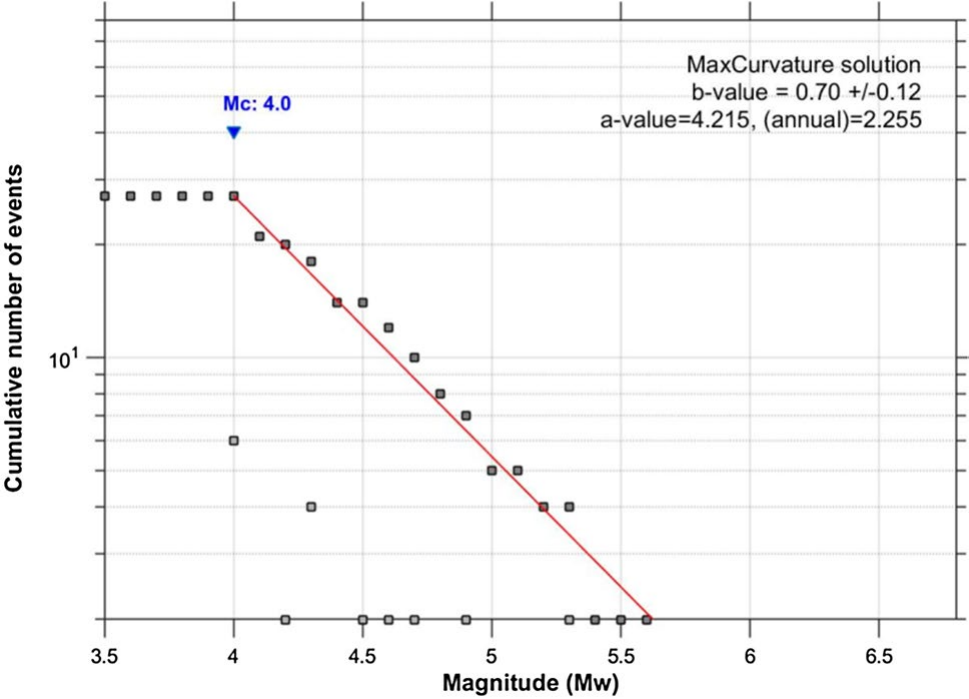
Frequency-Magnitude Distribution from 1818–2018 earthquake catalogue.

**Figure 5:**
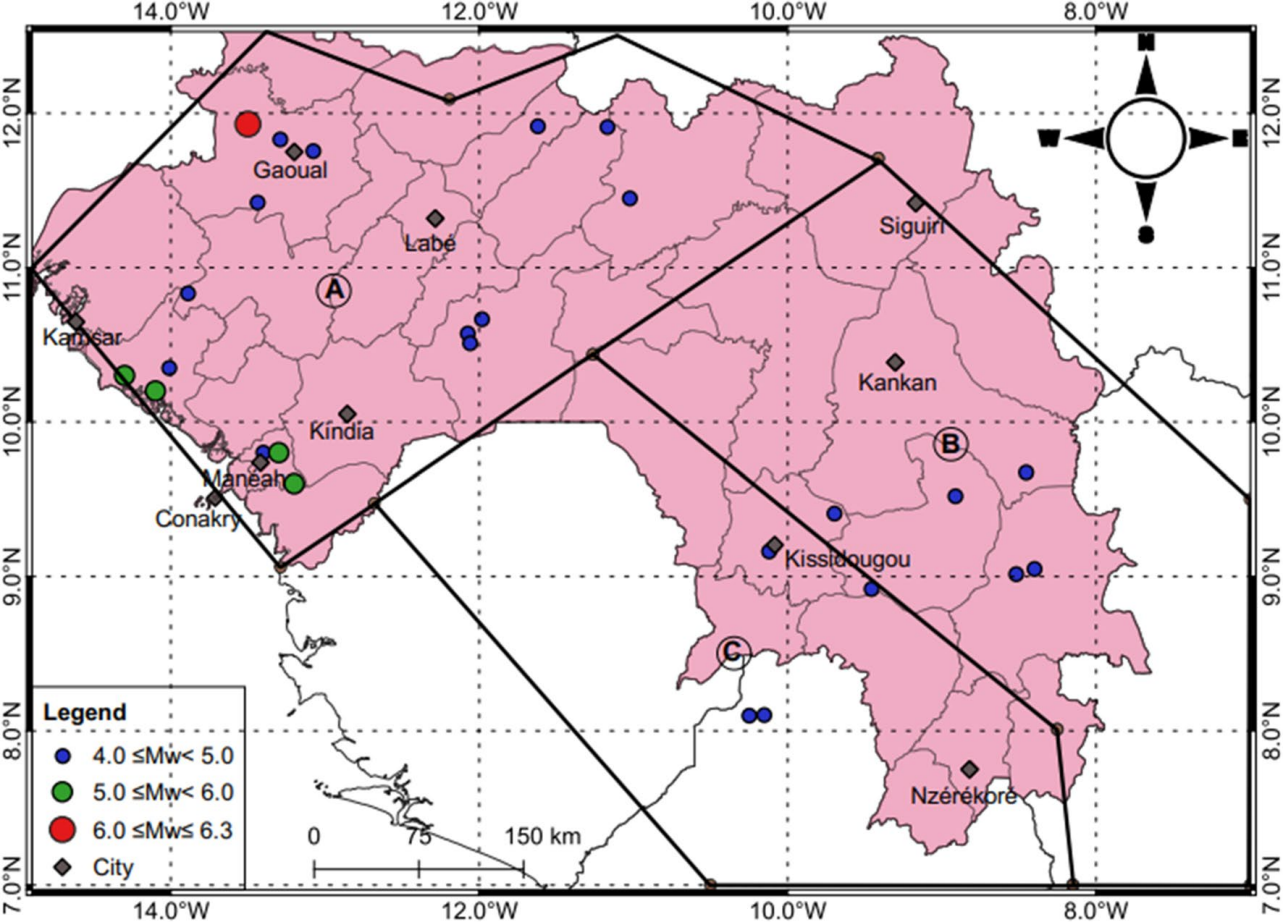
Seismic source zones for the study region.

Since information regarding the local faults for this study region is poorly documented; the determination of seismic hazard was based on available information on seismicity and geological settings of the study area. Three seismic source zones are used to estimate the hazard (see Figs. 4, 5). Zone A represents the Palaeozoic craton, while zones B and zone C, are represent the Archaean and Lower Proterozoic rocks.

In order to define the seismic source model, the three seismic source zones need to be characterised in terms of their earthquake recurrence, i.e., the relative frequency of occurrence of earthquakes of different sizes, as well as the maximum expected magnitude. These parameters were computed using the declustered catalogue discussed earlier. The seismicity of all seismic source zones is assumed to follow a truncated exponential (Gutenberg-Richter) distribution characterised by Eq. (6).

6 $$Log_{10} \left( N \right) \, = \, a - bM$$

where *N* = number of events with magnitudes equal to, or greater than, *M*, *a-*value = activity rate which defines Gutenberg-Richter relation intercept at *M* equal zero. The *b****-***value indicates the relative number of large and small earthquakes and represents the Gutenberg-Richter relation.

It is common to use a unique *b*-value for source zones in low-to-moderate seismicity due to limited recorded data^16,17^. As a result, a uniform *b*-value was calculated and adopted for all the zones (Table 2 and Fig. 4). The activity rate, (λ-parameter) is known to vary significantly for the different zones within a given area. It was estimated for each zone by taking the average number of earthquakes for magnitude equal to or higher than the minimum magnitude (*M*_min_). Out of the 27 events in the catalogue, 18 events are used for zone A, 5 events for zone B and 4 events for zone C respectively. We use maximum-likelihood method^11^ to estimate the recurrence parameters listed in Table 2.

**Table 2:** Recurrent parameters for each zone.

Zone	*M*_min_	*M*_*max*_	*b* ± *σb*	*a*	λ
A	4.0	6.8	0.70 ± 0.12	4.215	0.18
B	4.0	4.8			0.05
C	4.0	5.2			0.04

We estimate the maximum magnitude, *M*_max_ for each of the three seismic source zones. Many statistical methods are available to compute *M*_max_ (Kijko and Graham^18^; Kijko^19^). Owing to a lack of paleoseismic studies for the study area and the slow relative movement (~ 2 to ~ 15 mm/year) of the African Plate, which makes the recurrence period of strong earthquakes extremely long and thus unlikely to be included in our catalogue, we compute *M*_max_ by adding half magnitude increment to the maximum magnitude observed from the catalogue, such that *M*_max_ = 0.5 + *M*_obs_. This simplified method proposed by Gupta^20^ has been extensively used by other researchers for stable continental regions and where paleoseismic studies are limited, including studies focused on the seismicity of the Africa continent (Ahulu et al.^7^; Deif et al.^21^; Basir and Basu^22^; Poggi et al.^23^).

The accuracy of the focal depths is generally poor in this study region owing to the limited available information. However, based on the sources catalogue compiled for this study, the earthquake foci are within 10 to 15 km. Consequently, the hazard analyses were conducted using 10 and 15 km for all zones.

## Ground motion prediction equations

The ground-motion prediction equations (GMPEs) define the attenuation of ground motion amplitudes as a function of source-to-site distance and earthquake magnitude. In general, the selection of ground motion prediction equations (GMPEs) is the primary source of epistemic uncertainty in the seismic hazard analysis. We used two GMPES equations from a hybrid-empirical model developed for Eastern North America (ENA). ^24,25^ by assigning different weights for each seismic source zones using a logic-tree approach as discussed in the following section i. The equations for the GMPEs used in this study are listed in Table 3.

**Table 3:** List and conditions of GMPEs used in the study.

References	Ground motion prediction equations (GMPEs)	Salient features
Pezeshk et al.^24^	$\log \left( {\overline{Y}} \right) = c_{1} + c_{2} M_{w} + c_{3} M_{w}^{2} + \left( {c_{4} + c_{5} M_{w} } \right) \times min\left\{ {\log \left( R \right),\log \left( {60} \right)} \right\} + \left( {c_{6} + c_{7} M_{w} } \right) \times max\left[ {min\left\{ {\log \left( \frac{R}{60} \right),\log \left( {120/60} \right)} \right\},0} \right] { } + \left( {c_{8} + c_{9} M_{w} } \right) \times max\left\{ {\log \left( {R/120} \right),0)} \right\} + c_{10} R$ where $\left( {\overline{Y}} \right)$ is the median value of PGA or PSA in g, *R* is the distance computed as $R = \sqrt {R_{rup}^{2} + c_{11}^{2} }$ where *R*_*Rup*_ = closest distance to fault rupture in km, and *c*_1_ to *c*_11_ are regression coefficients as defined in Pezeshk et al.^25^	Based on hybrid empirical method (HEM). The GMPEs are derived for peak ground acceleration and response spectral ordinates at periods ranging from 0.01 to 10 s. Suitable for moment magnitudes (M_w_) from 4.0 to 8.0. Valid for *R*_*Rup*_ < 300–400 km. Mean aleatory standard deviation associated with the prediction is given by $\sigma_{T} = \sqrt {\sigma_{{{\text{log}}}}^{2} + \sigma_{{{\text{Reg}}}}^{2} }$ σ_Reg_ is the standard deviation of the regression. σ_logȲ_ is the total aleatory standard deviation. The values are given in Pezeshk et al.^24^^.^ Hard-rock site condition. *V*_S30_ = 3000 m/s
Tavakoli and Pezeshk^25^	${\text{Ln}}\left( Y \right){ } = f_{1} \left( {M_{w} } \right){ } + f_{2 } \left( {R_{rup} } \right) + f_{3} (M_{w} , R_{rup} ){ }R = { }\sqrt {R^{2}_{rup} + (C_{5 } {\text{exp}} [C_{6} M_{w} + C_{7 } \left( {8.5 - M_{w} } \right)^{2.5} ])^{2} }$ where *Y* represents median value of PGA/PSA in (g), $M_{w}$ represents moment magnitude, $R_{rup}$ represents rupture distance and means the closest distance to the fault rupture in (km). $f_{1}$ to $f_{3}$ are frequencies (Hz), while and *c*_5_ to *c*_7_ are the regression coefficients listed in Tavakoli and Pezeshk^25^	Based on a hybrid-empirical model is utilized to predict the ground-motion relationship for eastern North America (ENA). This is an empirical-stochastic attenuation relationship used for horizontal peak ground acceleration and for spectral acceleration. Applicable to *M*_w_ 5.0–8.2. *R*_*R*up_ $<$ 1000 km. Hard-rock site condition. *V*_S30_ = 2880 m/s. The aleatory standard deviation of ln *Y* is defined as a based on the earthquake magnitude and is modelled as follows

## Treatment of epistemic uncertainty

**Figure 6:**
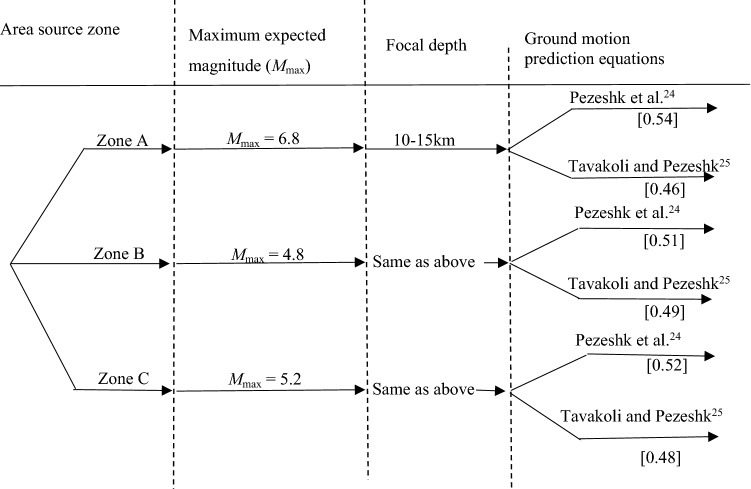
Logic-tree used for hazard calculation. The weights are in shown in parentheses.

We explicitly consider epistemic uncertainty using the logic tree approach ^26–29^. Each branch of the logic tree uses a different GMPE. Appropriate weight for each GMPEs are computed using the average sample log likelihood (LLH) function^30^, expressed as

7 $${\text{LLH }}\left( {g,{ }x_{i} } \right) = { } - { }\frac{1}{N}{ }\mathop \sum \limits_{i = 1}^{N} log_{2} \left[ {g\left( {x_{i} } \right)} \right]{ }$$

where $x_{1}$, $x_{2}$, $x_{3}$,…, $x_{N}$ are samples of the ground motion values determined from a GMPE model *g*($x_{i}$). The value of LLH (*g*, $x_{i}$) are used as a ranking criterion. The weight of each branch ($w_{i}$) can be calculated as:

8 $$w_{i} { } = { }{\raise0.7ex\hbox{${2^{{ - LLH_{i} }} }$} \!\mathord{\left/ {\vphantom {{2^{{ - LLH_{i} }} } {\mathop \sum \nolimits_{k = 1}^{K = M} 2^{{ - LLH_{k} }} }}}\right.\kern-\nulldelimiterspace} \!\lower0.7ex\hbox{${\mathop \sum \nolimits_{k = 1}^{K = M} 2^{{ - LLH_{k} }} }$}}$$

with *M* = 2. The GMPE ranking is calculated only on PGA value using each observation of $x_{i}$, 1, … *N,* which are 50 km, 100 km, 120 km, 150 km and 200 km*.* The sample log likelihood for the individual GMPE model is given $x_{i}$. The quantities have been averaged using Eq. (7) at (*N* = 5). The rankings of the GMPEs, are based on mean LLH_*i*_ values, and the weight for the individual GMPE is provided in Table 4 and in Fig. 6.

**Table 4:** Computed weights for different GMPEs for the three seismic source zones.

Zone	*M*_max_	TP2005	PEAL2018
A	6.8	0.46	0.54
B	4.8	0.49	0.51
C	5.2	0.48	0.52

### Hazard results and discussions

The seismic hazard calculations were performed using the R-CRISIS software, a probabilistic seismic hazard analyses software developed by II-UNAM, the Instituto de Ingeniería at UNAM, México^31^. The software is available for free on the R-CRISIS website. The analysis was conducted on a 0.5-degree grids spacing under the following conditions: Hazard calculations was computed for rock site conditions for 0.5%, 2%, and 10% probability of exceedance in 50 years, corresponding to return periods of 475, 2475, and 9975 years respectively. A minimum magnitude(*M*_min_) of 4.0 was adopted.

**Figure 7:**
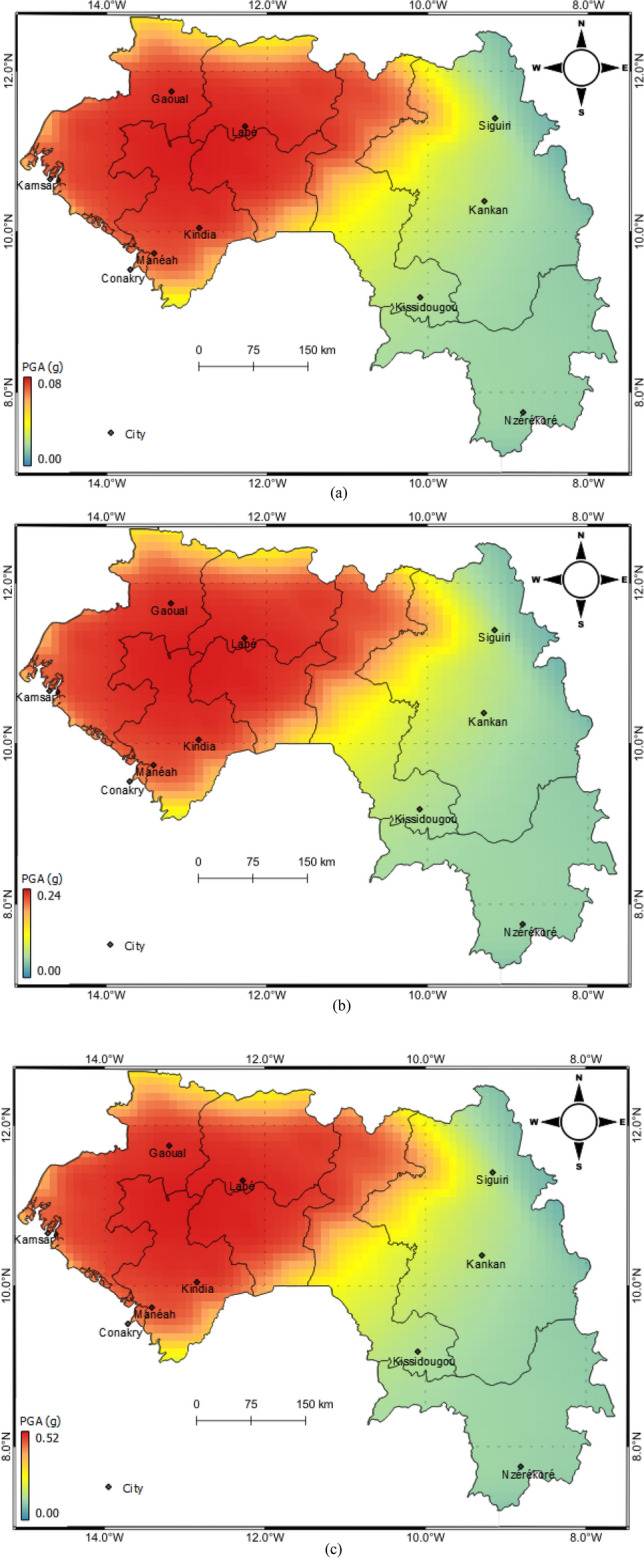
Seismic hazard maps at (**a**) 475-year (**b**) 2475-year (**c**) 9975-year, return periods.

Figure 7 shows the hazard maps in terms of mean PGA for 475, 2475, and 9975 years return periods. The results show that the highest level of seismic hazard is computed in northwest Guinea, where the maximum PGA values are 0.08 g, 0.27 g and 0.56 g at 475, 2475, and 9975 years return periods, respectively.

**Figure 8:**
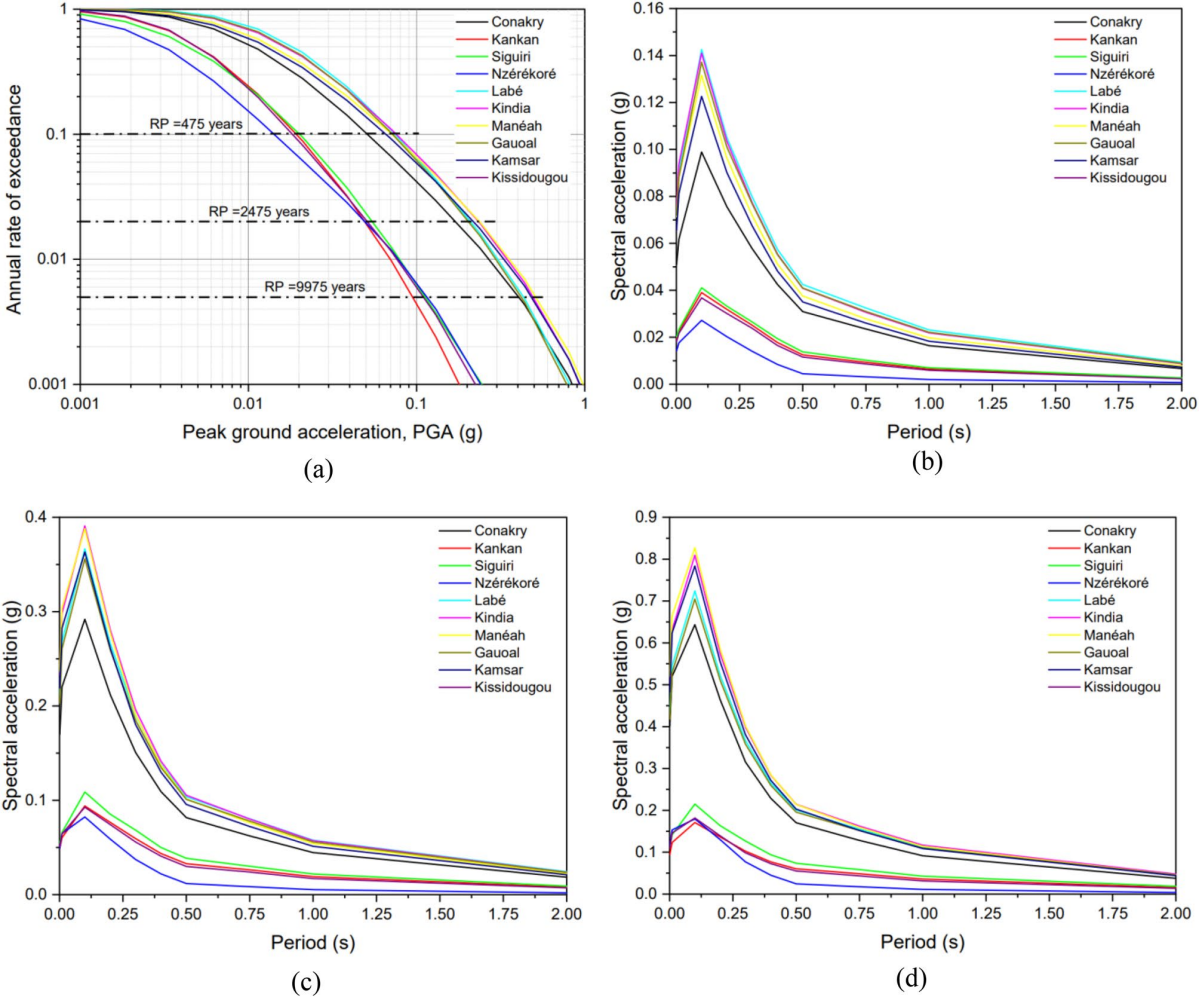
(**a**) Mean seismic hazard curves for PGA for ten analysed cities in Guinea. Uniform hazard spectra for selected ten cities within Guinea at (**b**) 475-year (**c**) 2475-years (**d**) 9975-year, return periods.

We also determined seismic hazard curves and uniform hazard spectra for ten cities in Guinea (see Fig. 8). Table 5 lists PGAs for the 475, 2475 and 9975 years return periods for the 10 selected cities. The cities of Labé (second largest city in Guinea), Gauoal and Kindia are located in the northwest part of the country and their higher seismic hazard is reflected by the high values as shown in the corresponding UHS (Fig. 8). The other cities have considerably lower seismic hazard, with the lowest PGA at Nzérékoré. Taking Conakry as an example, the largest city and capital of Guinea, the spectral acceleration (SA) at 0.1 s, is 0.11 g, 0.32 g and 0.71 g at 475, 2475 and 9975 years return periods, respectively.

**Table 5:** Peak ground acceleration (PGA) likely to be exceeded with probability of 10%, 2% and 0.5% corresponding to 475-year, 2475-year and 9975-year return periods, respectively for the selected sites within Guinea.

City	Latitude	Longitude	PGA (g)		
			475 Years	2475 Years	9975 Years
Conakry	9.509	− 13.712	0.055	0.184	0.445
Kankan	10.383	− 9.300	0.023	0.068	0.151
Siguiri	11.416	− 9.166	0.023	0.066	0.144
Nzérékoré	7.750	− 8.816	0.017	0.068	0.173
Labé	11.316	− 12.283	0.080	0.272	0.570
Kindia	10.049	− 12.854	0.080	0.268	0.570
Manéah	9.7333	− 13.416	0.078	0.261	0.565
Gauoal	11.750	− 13.200	0.080	0.270	0.570
Kamsar	10.650	− 14.616	0.072	0.241	0.534
Kissidougou	9.1833	− 10.100	0.022	0.070	0.169

**Figure 9:**
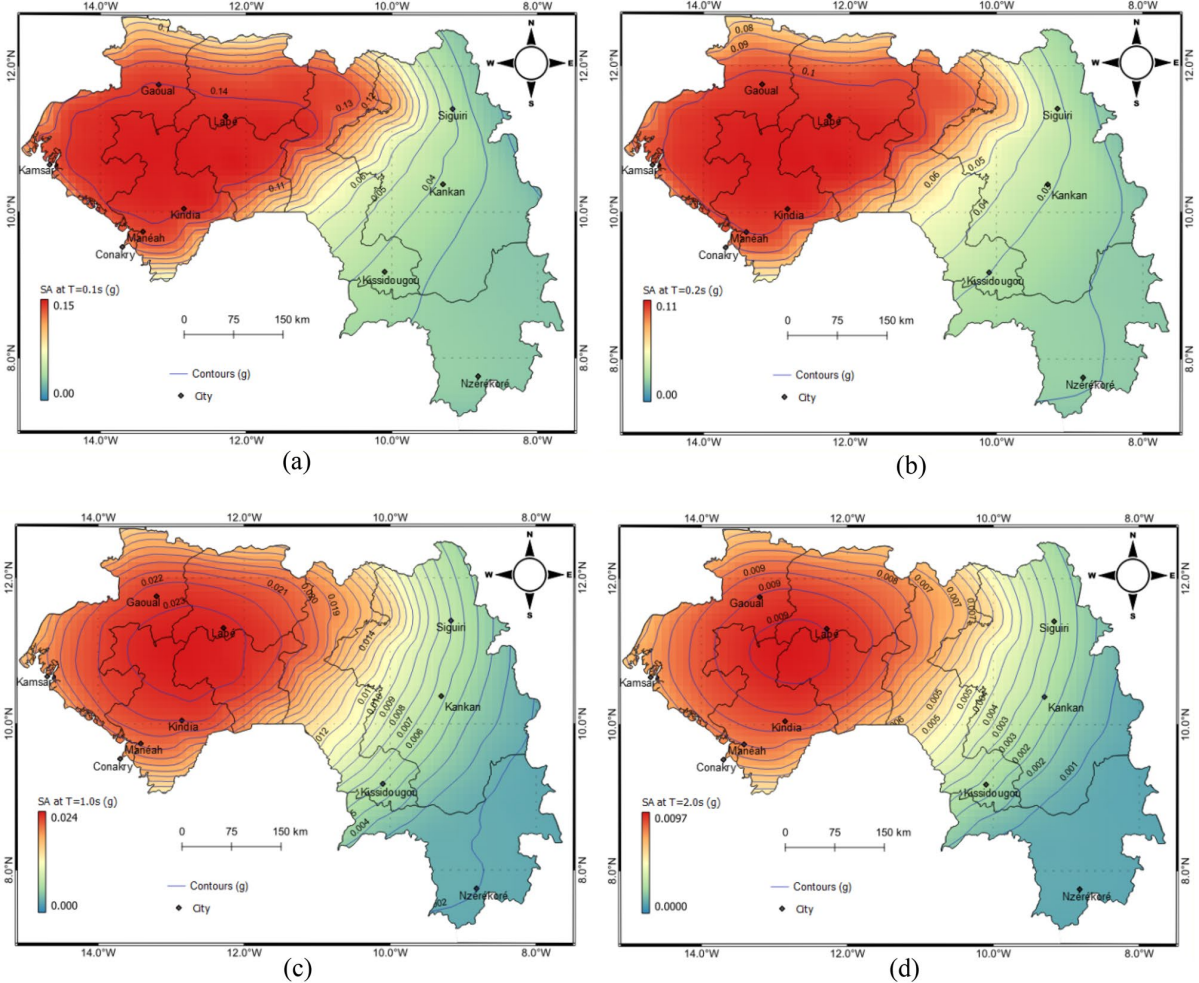
Seismic hazards on rock for spectral accelerations (SA) (**a**) 0.1, (**b**) 0.2, (**c**) 1.0 and (**d**) 2.0 s, respectively, (**g**) for 5% damping for a 475-year return period.

Figure 9 shows the contour of spectral accelerations (SA) at four different vibration periods 0.1 s, 0.2 s, 1.0 s and 2.0 s, 475 years return period, and bedrock conditions. The different vibrations periods are relevant to the seismic assessment and design of  different types of infrastructure (e.g., buildings, dams, bridges, etc.) since the level of damage due to the ground shaking depends on the proximity of the vibration period of the infrastructure to the predominant period of the earthquake^21^. The results show that the seismic hazard estimated for Guinea can be described as low to moderate at the sites selected. Based on these results, the following conclusions may be drawn:

- Major seismic hazard in Guinea is observed in the north-western part of the country, i.e., the Palaeozoic area, which has experienced some major earthquakes in the past.
- The seismic hazard is reduced in the Archaean and Lower Proterozoic areas as compared to the Palaeozoic area.
- The north-western part of Guinea where the city of Gauoal, the epicentre of earthquake of 1983, shows higher hazard values 0.16 g, 0.11 g, 0.02 and 0.008 for SA of 0.1 s, 0.2 s, 1.0 s and 2.0 s, respectively.

## Conclusion

A Probabilistic seismic hazard analysis for Guinea, West Africa, was performed based on a new compiled earthquake catalogue of duration of 100 years (1918–2018), estimated to be complete for magnitude *M*_w_ > 4. The catalogue was compiled using data from three sources, i.e.: the catalogue of Ambraseys and Adams^1^, ISC and USGS catalogues. The  new compiled catalogue was used to determine recurrence parameters and *M*_max_ values for three seismic source zones in which the study region was divided into. The boundaries of each seismic source zone were determined based on geology and seismicity of the region. Owing to a lack of strong ground motion records, region-specific GMPEs were not available. Therefore, the relations used in the study were adopted from those calibrated for the Eastern North America (ENA) region, which similar to the study region, is a stable continental region (SCR).

Seismic hazard maps for different return periods were produced for Guinea; while seismic hazard curves were computed for ten cities. The results showed that the seismic hazard is highest in cities located in the north-western part of the country. From this study, six out of ten cities (i.e., Gaoual, Labé, Kindia, Kamar, Manéah and Conakry) fall within the high hazard levels for the new seismic hazard maps. The hazard levels estimated are critical since the ten cities are now seeing sizeable infrastructure development. Therefore, the seismic risk associated with an increase in population and the expansion of the regional infrastructure can be significant. Thus, to protect the local population and to sustain the region’s economic development, it is critical to take into account this newly estimated heighten seismic risk, which, from an engineering design point of view, can be achieved by designing earthquake-resistant infrastructure and retrofitting existing buildings.
